# Improved Early-Phase Insulin Response after Candesartan Treatment in Hypertensive Patients with Impaired Glucose Tolerance

**DOI:** 10.1080/10641960802269927

**Published:** 2008-07-21

**Authors:** Katsunori Suzuki, Osamu Nakagawa, Yoshifusa Aizawa

**Affiliations:** ^1^Division of Endocrinology and Metabolism, Saiseikai Niigata Second Hospital, Niigata, Japan; ^2^Division of Endocrinology and Metabolism, Sanjo General Hospital, Sanjo, Niigata, Japan; ^3^Division of Cardiology, Niigata University Graduate School of Medical and Dental Sciences, Niigata, Japan

**Keywords:** candesartan, IGT, insulinogenic index, insulin secretion

## Abstract

As the effect of renin-angiotensin system (RAS) blockade on β-cells in clinical situations remains unclear, new evidence has been presented that angiotensin-converting enzyme (ACE) inhibitors and angiotensin ‖ receptor blockers (ARBs) may delay or prevent the development of insulin resistance and diabetes through novel mechanisms. This study aimed to determine the effects of ARBs on insulin excretion by β-cells. Hypertensive patients with impaired glucose tolerance were randomly divided into two groups: group A (n = 6), which received 8 mg/day of oral candesartan for three months, and controls (n = 6). Before and after administration, a 75 g oral glucose tolerance test was conducted to compare various parameters. No significant differences in age, body mass index (BMI), systolic blood pressure (SBP), diastolic blood pressure (DBP), fasting glucose, or fasting immunoreactive insulin (IRI) were identified between the groups before administration. After three months, there were no significant changes in BMI, SBP, and DBP for the controls and in BMI and DBP for group A. However, SBP was significantly decreased from 144 ± 2.6 mmHg to 125 ± 4.6 mmHg in group A. Insulinogenic index tended to be slightly decreased for controls, but was significantly increased from 0.32 ± 0.0 to 0.47 ± 0.1 for group A. No significant changes in HOMA-R were identified in either group. To the best of our knowledge, no previous studies have documented a RAS inhibitor improving early-phase insulin response; thus, the present study may be the first of its kind.

## Introduction

Recent trials have suggested that inhibitors of the renin-angiotensin system (RAS), such as angiotensin-converting enzyme (ACE) inhibitors and angiotensin ‖ receptor blockers (ARBs), may reduce the incidence of new-onset of diabetes in patients with or without hypertension and at a higher risk of developing diabetes (1). This reduction has been explained by hemodymic effects, such as improved delivery of insulin and glucose to peripheral skeletal muscle, and nonhemodynamic effects, such as direct effects on glucose transport and insulin signaling pathways, all of which decrease insulin resistance (2,3). There is some evidence now to suggest that the pancreas may contain an in situ active RAS, which appears to be upregulated in animal models of type 2 diabetes (4,5). ACE inhibitors and ARBs may thus act by attenuating the deleterious effects of angiotensin ‖ on vasoconstricton, fibrosis, inflammation, apoptosis and β-cell death in the pancreas, thereby protecting critical β-cell mass essential for insulin production. Therefore, these findings provide a novel mechanism focusing on the β-cell that could partly explain the reduced incidence of new-onset diabetes observed in clinical trials involving therapy with ACE inhibitors or ARBs. This study aimed to determine the effects of candesartan on insulin excretion by β-cells after oral glucose loading in hypertensive patients with impaired glucose tolerance (IGT).

## Research Design and Methods

Hypertensive patients with IGT were enrolled in this study at Saiseikai Niigata Second Hospital. None of the enrolled patients with IGT had received pharmacologic treatment before the study. Hypertension was defined by systolic blood pressure (SBP) ≥140 mmHg and/or diastolic blood pressure (DBP) ≥90 mmHg. A standard oral glucose tolerance test (OGTT) with 75 g of glucose was performed before and after administration. Interpretations of OGTT data were based on the World Health Organization definition (6), and the diagnostic criteria for IGT were included in the American Diabetes Association criteria (7). Insulin resistance expressed as the homeostasis model assessment for insulin resistance (HOMA-R) was calculated under fasting conditions as follows:

plasma insulin (μU/ml)×blood glucose (mg/dl)/405 (8).

Insulinogenic index is equal to the increase in insulin secretion (immunoreactive insulin IRI at 30 min - IRI at 0 min) divided by the increase in plasma glucose (plasma glucose at 30 min - plasma glucose at 0 min) (9). Patients were randomly divided into two groups: controls (n = 6), who were only given dietary guidance, and group A (n = 6), who were receiving 8 mg/day of oral candesartan for three months in addition to dietary guidance. All participants (controls+group A) were in a normal dietary education program and encouraged to exercise regularly during the study. Numerical data are expressed as mean ± standard error of the mean. Wilcoxon signed rank sum tests were used for statistical analyses. Informed consent to participate in the study was obtained from each patient. All study protocols were approved by independent local institutional review boards.

## Results

**Figure 1:**
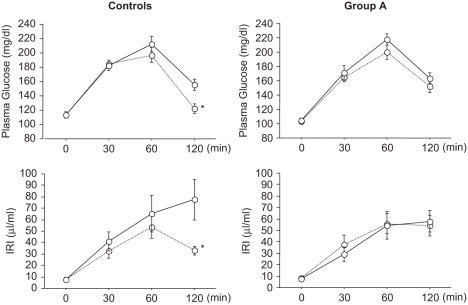
Time changes in plasma glucose and IRI after glucose loading before and after three months for the controls and group A. Solid line = baseline values; dotted line = post-administration values. Differences between two paired variables were analyzed using the Wilcoxon single-rank test. *p < 0.05.

**Figure 2:**
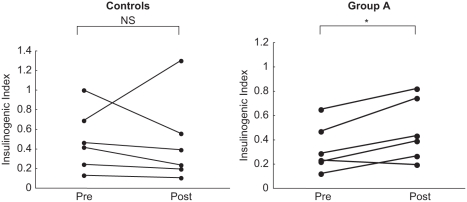
Individual changes in insulinogenic index before and after three months for the controls and group A. Differences between two paired variables were analyzed using the Wilcoxon single-rank test. *p < 0.05.

Table 1 shows the clinical characteristics in controls and group A. No significant differences in age, body mass index (BMI), SBP, DBP, fasting glucose, fasting IRI, or lipid profile were identified between the groups before administration. After three months, there was no significant change in BMI, SBP, and DBP in the controls, or in BMI and DBP in group A (see Table 2). However, SBP was significantly decreased from 144 ± 2.6 mmHg to 125 ± 4.6 mmHg in Group A. Figure 1 shows changes in IRI and plasma glucose as measured by a OGTT before and after glucose administration. In the controls, both plasma glucose level and IRI were significantly decreased after 120 min. In group A, IRI was slightly increased at 30 min, and did not decrease thereafter. Insulinogenic index tended to be slightly decreased for the controls, but was significantly increased from 0.32 ± 0.0 to 0.47 ± 0.1 for group A (see Table 2). In terms of individual changes, insulinogenic index decreased in all the control patients except for one, and increased in all patients in group A except for one (see Figure 2). No significant changes in HOMA-R were identified in either group (see Table 2). During the study period, normal glucose tolerance was regained in two patients in each group, and no patients in either group developed type 2 diabetes.

**Table 1:** Basal characteristics in the controls and group A

	Controls	Group A
N	6	6
Age (y)	53.1 ± 5.1	57.6 ± 4.9
BMI (Kg/m^2^)	25.0 ± 1.4	25.5 ± 2.0
SBP (mmHg)	140.3 ± 2.7	144.3 ± 2.6
DBP (mmHg)	80.6 ± 3.5	85.3 ± 1.9
Fasting glucose (mg/dl)	112 ± 4.4	105 ± 2.6
Fasting IRI (μU/ml)	7.9 ± 1.7	7.6 ± 0.9
Total cholesterol (mg/dl)	169.5 ± 6.1	168.8 ± 6.4
Triglycerides (mg/dl)	121.5 ± 21	107.3 ± 17
HDL cholesterol (mg/dl)	45.0 ± 3.6	48.1 ± 3.1
LDL cholesterol (mg/dl)	101.0 ± 6.4	99.3 ± 6.3

Values are expressed as number or mean ± standard error of the mean (SEM). No significant differences in any parameters were identified between the groups at baseline.

Abbreviations: BMI = body mass index, SBP = systolic blood pressure, DBP = diastolic blood pressure, IRI = immunoreactive insulin.

**Table 2:** Changes in clinical parameters before and after three months for the controls and group A

	Controls		Group A	
		
	Pre	3M	Pre	3M
BMI (Kg/m^2^)	25.0 ± 1.4	24.5 ± 1.3	25.5 ± 2.0	25.8 ± 2.1
SBP (mmHg)	140.3 ± 2.7	137.0 ± 1.5	144.3 ± 2.6	125.6 ± 4.6*
DBP(mmHg)	80.6 ± 3.5	79.7 ± 3.3	85.3 ± 1.9	83.0 ± 1.3
FPG(mg/dl)	112 ± 4.4	113 ± 3.7	105 ± 2.6	103 ± 3.7
FIRI (μU/ml)	7.9 ± 1.7	7.8 ± 1.6	7.6 ± 0.9	8.3 ± 0.4
AUC for PG (mg/dl·min)	21412.5 ± 731	19767.5 ± 655*	21432.5 ± 784	20080.0 ± 719
AUC for IRI (μU/ml·min)	6622.2 ± 1469	4501.0 ± 677	5175.5 ± 940	5395.2 ± 676
HOMA-R	2.18 ± 0.5	2.17 ± 0.4	1.97 ± 0.2	2.16 ± 0.1
Insulinogenic	0.48 ± 0.1	0.46 ± 0.1	0.32 ± 0.0	0.47 ± 0.1*

Values are expressed as mean ± SEM.

Abbreviation: AUC = the area under the curve.

* *p* < 0.05 vs. baseline data for each group.

## Conclusions

The present results show that three months of candesartan therapy improved early-phase insulin response in IGT patients with hypertension. RAS blockade in animal models of type 2 diabetes significantly restores β-cell mass, which could be interpreted as representing a potentially reparative mechanism, possibly by decreasing oxidative stress and apoptosis, in addition to attenuating profibrotic pathways (4,5,10). These findings indicate a novel mechanism focusing on β-cells that could partially explain the reduced incidence of new-onset of diabetes observed in clinical trials involving therapy with ACE inhibitors or ARBs. Although the details of the mechanisms of action could not be clarified, candesartan could improve insulin resistance (11–13) and thus relieve glucotoxicity, improving insulin secretion. Another possibility is that candesartan acts directly on pancreatic β-cells to improve early-phase insulin response. In the present study, no changes were noted in HOMA-R, an insulin resistance marker, suggesting that the latter mechanism is mainly responsible. For reason why HOMA-R was not changed after the administration of candesartan, we speculate that candesartan did not improve clinical insulin sensitivity because the study period was relatively short (i.e., six months). To the best of our knowledge, no previous studies have documented a RAS inhibitor improving early-phase insulin response. Thus, the present study is the first of its kind. Subjects in the present study displayed IGT where insulin secretion was relatively maintained, and a similar study is needed in patients with type 2 diabetes where insulin secretion is somewhat depressed.

Major limitations of the present study were that we examined only a small number of patients and the study period was relatively short (i.e., six months). Therefore, larger studies are needed to further elucidate the insulin secretory effect of candesartan during long-term follow-up.
